# Supplementary material for: Distinct inflammatory and wound healing responses to complex caudal fin injuries of larval zebrafish
Source: eLife. 2019 Jul 1;8:e45976. doi: 10.7554/eLife.45976 (PMC6602581; doi:10.7554/eLife.45976)
Supplement: Figure 1—source code 1. [file elife-45976-fig1-code1.docx]

**Figure 1 source code 1:** SAS code for repeated measure data for Figure 1D, E

options nocenter ls=**132** ps=**70**;

**data** a;

input rep time cond $ larva measure;

datalines;

**proc** **print**;

**run**;

**proc** **univariate** noprint;

histogram measure;

**run**;

**proc** **mixed**; class rep cond larva time;

model measure = cond time cond*time / outp=newfile;

random rep larva(rep*cond);

repeated / subject=larva(rep*cond) type=ar(**1**);

lsmeans cond time cond*time / diff cl;

ods output diffs=ppp lsmeans=mmm;

ods listing exclude diffs lsmeans;

**run**;

%include 'I:\pdmix800.sas';

%***pdmix800***(ppp,mmm,alpha=**.05**,sort=yes);

**run**;

**proc** **univariate** data=newfile plot normal;

var resid;

**run**;

**proc** **rank** data=a out=b;

var measure;

ranks rmeasure;

**run**;

**proc** **print** data=b;

**run**;

**proc** **mixed** data=b; class rep cond time;

model rmeasure = cond time time*cond;

random rep rep*cond;

lsmeans cond time cond*time / diff cl;

**run**;

**proc** **mixed** data=b; class rep cond larva time;

model rmeasure = cond time cond*time;

random rep larva(rep*cond);

repeated / subject=larva(rep*cond) type=ar(**1**);

lsmeans cond time cond*time / diff cl;

ods output diffs=ppp lsmeans=mmm;

ods listing exclude diffs lsmeans;

**run**;

%include 'I:\pdmix800.sas';

%***pdmix800***(ppp,mmm,alpha=**.05**,sort=yes);

**run**;

NOTE: pdmix800 is a macro freely available for SAS, copyright 2000 Arnold M. Saxton ([asaxton@utk.edu](mailto:asaxton@utk.edu)) University of Tennessee, Knoxville, TN.
