# Supplementary material for: Distinct inflammatory and wound healing responses to complex caudal fin injuries of larval zebrafish
Source: eLife. 2019 Jul 1;8:e45976. doi: 10.7554/eLife.45976 (PMC6602581; doi:10.7554/eLife.45976)
Supplement: Figure 2—source code 2. [file elife-45976-fig2-code2.docx]

**Figure 2 source code 2:** SAS code for count data, Poisson distribution for Figure 2F

options nocenter ls=**132** ps=**70**;

**data** a;

input

rep cond $ time measure;

datalines;

**proc** **print**;

**run**;

**proc** **univariate** noprint;

var measure;

histogram measure;

**run**;

**Proc** **GLIMMIX**;

class rep cond time;

model measure = cond time cond*time/dist=poi link=log;

random rep rep*cond;

lsmeans cond time time*cond/diff cl ilink;

run;
