# Supplementary material for: Distinct inflammatory and wound healing responses to complex caudal fin injuries of larval zebrafish
Source: eLife. 2019 Jul 1;8:e45976. doi: 10.7554/eLife.45976 (PMC6602581; doi:10.7554/eLife.45976)
Supplement: Figure 2—source code 3. [file elife-45976-fig2-code3.docx]

**Figure 2 source code 3:** SAS code LSMeans analysis plus rank analysis Figure 2G

options nocenter ls=**132** ps=**70**;

**data** a;

input

rep cond $ time measure;

datalines;

**proc** **print**;

**run**;

**proc** **univariate** noprint;

histogram measure;

**run**;

**proc** **mixed**; class rep cond time;

model measure = cond time time*cond/outp=newfile;

random rep rep*cond;

lsmeans cond time cond*time / diff cl;

lsmeans time*cond / slice=time cl;

lsmeans time*cond / slice=cond cl;

**run**;

**proc** **univariate** data=newfile plot normal;

var resid;

**run**;

**proc** **rank** data=a out=b;

var measure;

ranks rmeasure;

**run**;

**proc** **print** data=b;

**run**;

**proc** **mixed** data=b; class rep cond time;

model rmeasure = cond time time*cond;

random rep rep*cond;

lsmeans cond time cond*time / diff cl;

lsmeans time*cond / slice=time cl;

**run**;
