# Supplementary material for: Distinct inflammatory and wound healing responses to complex caudal fin injuries of larval zebrafish
Source: eLife. 2019 Jul 1;8:e45976. doi: 10.7554/eLife.45976 (PMC6602581; doi:10.7554/eLife.45976)
Supplement: Figure 3—source code 1. [file elife-45976-fig3-code1.docx]

**Figure 3 source code 1:** ImageJ macro for measuring area devoid of SHG fibers for Figure 3 B, D

//macro expects a merged BF and Fluorescent image

Dialog.create("Please Select Merged BF and Fluorescent Image");

Dialog.addMessage("Please Select Merged BF and Fluorescent Image");

Dialog.show();

mergeImage=File.openDialog("Merged image");

open(mergeImage);

mergeImageName=getTitle;

dir = getDirectory("image");

run("Split Channels");

open(mergeImageName);

selectWindow("C2-" + mergeImageName);

setTool("zoom");

Dialog.create("Click image to zoom");

Dialog.addMessage("Click image to zoom");

Dialog.show();

waitForUser("image zoomed");

Dialog.create("Adjust intensity");

Dialog.addMessage("Adjust intensity");

Dialog.show();

run("Brightness/Contrast...");

waitForUser("image adjusted");

Dialog.create("Draw line at fiber ends");

Dialog.addMessage("Draw line at fiber ends");

Dialog.show();

setTool("freeline");

waitForUser("Use this selection");

getStatistics(length)

roiManager("Add");

selectWindow(mergeImageName);

roiManager("Select", 0);

run("Flatten");

run("Flatten");

setTool("zoom");

Dialog.create("Click image to zoom");

Dialog.addMessage("Click image to zoom");

Dialog.show();

waitForUser("image zoomed");

setTool("freehand");

Dialog.create("Draw area from fiber ends to wound edge");

Dialog.addMessage("Draw area from fiber ends to wound edge");

Dialog.show();

waitForUser("Use this selection");

getStatistics(area);

run("Flatten");

currRow = nResults;

setResult("File Name", currRow, mergeImageName);

setResult("fiber edge line area",currRow, length);

setResult("Tissue Area", currRow, area);

updateResults();

saveAs("Tiff", dir + "/flat_" + mergeImageName);

roiManager("Deselect");

roiManager("Delete");

close();

close();

close();

close();

close();
