# Supplementary material for: Distinct inflammatory and wound healing responses to complex caudal fin injuries of larval zebrafish
Source: eLife. 2019 Jul 1;8:e45976. doi: 10.7554/eLife.45976 (PMC6602581; doi:10.7554/eLife.45976)
Supplement: Figure 3—source code 3. [file elife-45976-fig3-code3.docx]

**Figure 3 source code 3:** ImageJ macro for counting GFP cells in a designated ROI for Figure 3G

Dialog.create("select file to open");

Dialog.addMessage("Please Select Image to count");

Dialog.show();

countImage=File.openDialog("Count Image");

open(countImage);

countImageName=getTitle;

dir = getDirectory("image");

Dialog.create("Adjust intensity");

Dialog.addMessage("Adjust intensity");

Dialog.show();

run("Brightness/Contrast...");

waitForUser("image adjusted");

setTool("rectangle");

makeRectangle(0, 0, 120, 240);

Dialog.create("move rectangle");

Dialog.addMessage("move rectangle to desired location");

Dialog.show();

waitForUser("Rectangle is in the correct location");

run("Flatten");

Dialog.create("actual area");

Dialog.addMessage("Draw actual area used");

Dialog.show();

setTool("polygon")

waitForUser("Use selected area");

getStatistics(area)

run("Flatten");

saveAs("Tiff", dir + "/flat_" + countImageName);

setTool("zoom");

Dialog.create("Click image to zoom");

Dialog.addMessage("Click image to zoom");

Dialog.show();

waitForUser("image zoomed");

setTool("multipoint");

Dialog.create("Select point");

Dialog.addMessage("Use pointer to count cells. Alt-click deletes points");

Dialog.show();

waitForUser("count these points");

run("Properties... ", "name=filename show");

getSelectionCoordinates(xCoordinates, yCoordinates);

run("Flatten");

saveAs("Tiff", dir + "/count_flat_" + countImageName);

Dialog.create("Close Count results window");

Dialog.addMessage("Close count results window");

Dialog.show();

waitForUser("window closed");

currRow = nResults;

setResult("File Name", currRow, countImageName);

setResult("area measured",currRow, area);

setResult("GFP cells", currRow, xCoordinates.length);

updateResults();

close();

close();

close();

close();

Dialog.create("Do you want to count another image?");

Dialog.addMessage("Do you want to do another image?");

Dialog.show();

runMacro "<go to location of original macro to repeat>”);
