# Supplementary material for: Distinct inflammatory and wound healing responses to complex caudal fin injuries of larval zebrafish
Source: eLife. 2019 Jul 1;8:e45976. doi: 10.7554/eLife.45976 (PMC6602581; doi:10.7554/eLife.45976)
Supplement: Figure 3—source code 4. [file elife-45976-fig3-code4.docx]

**Figure 3 source code 4:** SAS code for count data, negative binomial distribution for Figure 3G

options nocenter ls=**132** ps=**70**;

**data** a;

input

rep cond $ time measure;

datalines;

**proc** **sort** data=a;

by time;

ods listing close;

**Proc** **GLIMMIX** data=a;

class rep cond;

model measure = cond rep /dist=NB;

lsmeans cond /pdiff adjust=Tukey ilink cl;

ods output Diffs=b LSMeans=c;

by time;

**run**;

ods listing;

**data** b;

set b;

pvalue=AdjP;

**proc** **print** noobs;

var time cond _cond pvalue;

**run**;

**data** c;

set c;

**proc** **print** noobs;

var time Cond Mu LowerMu UpperMu;

**run**;
