# Supplementary material for: Distinct inflammatory and wound healing responses to complex caudal fin injuries of larval zebrafish
Source: eLife. 2019 Jul 1;8:e45976. doi: 10.7554/eLife.45976 (PMC6602581; doi:10.7554/eLife.45976)
Supplement: Figure 4—source code 2. [file elife-45976-fig4-code2.docx]

**Figure 4 source code 2:** SAS code for count data with negative binomial distribution Figure 4D, H

options nocenter ls=**132** ps=**70**;

**data** a;

input

rep cond $ measure;

datalines;

**proc** **print**;

**run**;

**proc** **univariate** noprint;

histogram measure;

**run**;

**Proc** **GLIMMIX** data=a;

class rep cond;

model measure = cond rep cond*rep/dist=NB;

lsmeans cond /pdiff cl ilink;

**run**;
